# G Protein-Coupled Receptor Kinase 2 (GRK2) Regulates T Cell Response in a Murine Model of House Dust Mite-Induced Asthma

**DOI:** 10.3389/falgy.2021.656886

**Published:** 2021-05-17

**Authors:** Ananth K. Kammala, Canchai Yang, Reynold A. Panettieri, Rupali Das, Hariharan Subramanian

**Affiliations:** ^1^Department of Physiology, Michigan State University, East Lansing, MI, United States; ^2^Rutgers Institute for Translational Medicine and Science, New Brunswick, NJ, United States

**Keywords:** G protein-coupled receptor kinase 2, GRK2, asthma, airway inflammation, airway hyperresponsiveness, allergic diseases, goblet cell hyperplasia, house dust mite extract

## Abstract

G protein-coupled receptor kinase 2 (GRK2) is an adapter protein that modulates G protein-coupled receptor (GPCR) signaling. It also regulates the functions and activity of other intracellular proteins in many cell types. Accordingly, GRK2 is thought to contribute to disease progression by a variety of mechanisms related to its multifunctional roles. Indeed, GRK2 levels are enhanced in patient samples as well as in preclinical models of several diseases. We have previously shown that GRK2 regulates mast cell functions, and thereby contributes to exacerbated inflammation during allergic reactions. In the current study, we observed that GRK2 levels are enhanced in the lungs of human asthma patients and in mice sensitized to house dust mite extract (HDME) allergen. Consistent with these findings, interleukin (IL)-4 and IL-13 levels were reduced in the lungs of GRK2^+/−^ mice in a HMDE mouse model of asthma. Because Th2 cells are the major source of these cytokines during asthma, we determined the role of GRK2 in regulating T cell-specific responses in our HMDE mouse model. We observed a significant reduction of airway hyperresponsiveness (AHR), lung eosinophil and lymphocyte counts, serum IgE, Th2 cytokines (IL-4 and IL-13), goblet cell hyperplasia and mucus production in mice that had reduced GRK2 expression specifically in T cells. Collectively, our studies reveal an important role for GRK2 in regulating T cell response during asthma pathogenesis and further elucidation of the mechanisms through which GRK2 modulates airway inflammation will lead to the development of new therapeutic strategies for asthma.

## Introduction

Allergic asthma is a chronic inflammatory disease that is characterized by reversible airflow obstruction, mucosal inflammation, and airway hyperresponsiveness (AHR). Asthma patients generally have symptoms such as wheezing, coughing, chest tightness, and dyspnea, and if the disease is not treated, it may cause respiratory failure. It is well-established that type 2 (T2) allergic asthma is mediated by naïve CD4 T cells that mature into effector T cells when stimulated by allergens or antigens (1). These cells then produce Th2 cytokines such as IL-4 and IL-13 that causes B cells to produce IgE antibodies. The crosslinking of IgE receptors by these IgE antibodies and allergen causes mast cell activation and release of proinflammatory mediators (2). Additionally, other immune cells such as eosinophils and lymphocytes migrate to the lungs and regulate airway inflammation. The asthma pathology is amplified by airway obstruction due to mucus production, goblet cell hyperplasia and remodeling of airways by the airway epithelial and smooth muscle cells (3). Although the pathogenesis of asthma is complex and involves multiple cell types, T cells have been shown to play a critical role in allergic asthma induction. The mainstay therapy for asthma includes corticosteroids that affect T cell responses and humanized antibodies that inhibit the Th2 cytokines (4). However, there are subjects whose disease is not controlled by these agents and they account for the major healthcare costs of asthma, highlighting an urgent need for the development of new and effective therapies.

GRK2 is a member of a large family of GPCR kinases that is ubiquitously expressed in various cell types. GRK2 is important for desensitization of several GPCRs (5). The pivotal role of GRK2 in regulating GPCRs in the context of cardiac function is well-established and GRK2 is a major therapeutic target for cardiac disease (6, 7). Following ligand stimulation of GPCRs, GRK2 is recruited from the cytoplasm to the membrane and phosphorylates agonist-occupied receptors, abolishing G protein activation. Additionally, GRK2 can function as an adapter molecule and regulate cellular responses independent of GPCRs (8). Specifically, GRK2 has been shown to interact with intracellular signaling molecules and in some cases phosphorylate them (9). For example, GRK2 can phosphorylate P38 kinase to reduce lipopolysaccharide-induced macrophage activation (10). Conversely, other kinases such ERK1/2 and Src can phosphorylate GRK2 and affect its functions (11–13). We have previously demonstrated that GRK2 promotes IgE receptor induced mast cell activation, independent of its kinase function (14). GRK2 can thus regulate cellular responses induced by GPCRs and/or other receptors via phosphorylation-dependent and -independent mechanisms. Due to its diverse effects, GRK2 has been shown to play a critical role in various inflammatory diseases such as colitis, sepsis atherosclerosis, arthritis, and autoimmune encephalomyelitis (15).

Several reports have highlighted the role of GRK2 in modulating GPCR signaling in the airway epithelial (AEC) and smooth muscle cells (ASMC). GRK2 mediates internalization and desensitization of the β-2-adrenoceptor (β_2_AR) (16–18) that is expressed in AECs (19, 20). In addition, agonist-specific desensitization of the β_2_AR in ASMC cultures was reversed by siRNA-mediated knockdown of GRK2, or via expression of the dominant negative mutant of GRK2 (21). Interestingly, Albano et al. (22) reported that the desensitization of the β_2_AR in AECs by IL-13 is mediated via the GRK2 pathway. GRK2 also regulates the chemotaxis of cells given that most chemokine receptors are GPCRs. Accordingly, GRK2^+/−^ T cells show increased chemotaxis to the chemokines CCL3, CCL4, and CCL5 (23). Moreover, GRK2 promotes T cell receptor (TCR) signaling and is required for production of cytokines such as IL-2 and IL-10 following TCR activation (24). Though these findings underscore the role of GRK2 in regulating responses of different cell types involved in allergic airway disease, to our knowledge there has been no study till date that has examined the role of this adapter molecule in mediating the pathophysiology of asthma.

Using GRK2^+/−^ mice (GRK2^−/−^ is embryonically lethal), in the current study, we asked whether GRK2 regulated the development of asthma symptoms induced by HMDE. Exposure of wild type GRK2^+/+^ mice to HDME elicited a Th2 airway inflammatory response including AHR, lung immune cell infiltration, high levels of serum IgE, and Th2 cytokines (IL-4 and IL-13) in the lungs. While both IL-4 and IL-13 levels were reduced in GRK2^+/−^ mice, AHR and other parameters of airway inflammation were unaltered. To specifically dissect the contribution of GRK2 in regulating T cell responses during asthma, we generated the CD4 Cre^+^ GRK2^fl/fl^ mice (that lacked GRK2 in T cells) and exposed these mice to HDME. We observed a significant reduction of AHR, lung eosinophil and lymphocyte count, serum IgE, Th2 cytokines (IL-4 and IL-13), goblet cell hyperplasia and mucus production in the CD4 Cre^+^ GRK2^fl/fl^ mice as compared to the control CD4 Cre^−^ GRK2^fl/fl^ mice. Our data thus suggests that GRK2 plays an important role in promoting T lymphocyte responses in asthma.

## Materials and Methods

### Mice

C57BL/6J (B6), GRK2^fl/fl^, and CD4 Cre^+^ mice were obtained from The Jackson Laboratory (Bar Harbor, ME). GRK2^+/−^ mice were kindly provided by Dr. Narayanan Parameswaran [Michigan State University (MSU)]. Mice were bred and housed under specific pathogen-free conditions. The CD4 Cre^+^ GRK2^fl/fl^ (Cre^+^) mouse strain that lacks GRK2 in T cells were generated by crossing the CD4 Cre^+^ mice with the GRK2^fl/fl^ mice. Littermate CD4 Cre^−^ GRK2^fl/fl^ (Cre^−^) mice were used as controls. Six to eight-week-old male and female mice used for all the experiments that were approved by the Institutional Animal Care and Use Committee at MSU.

### Human Lung Samples

Lung samples from deceased subjects that died of asthma or other causes were obtained from either the International Institute for the Advancement of Medicine or National Disease Resource Interchange. All lung samples were harvested anonymously and de-identified. The use of these samples was approved by Institutional Review Board at Rutgers University. The available information for individual donors is provided in Supplemental Table 1.

### Mouse Model of Asthma

Mice were instilled intranasally (i.n) with either 25μl of HDME (50 μg of protein, Greer Laboratories, Lenoir, NC) or 25 μl of PBS on alternate days for a total of seven injections. The endotoxin level of the HDME preparation was 38.4 EU per injection. Twenty-four hours after the final HDME challenge, mice were anesthetized for measurement of AHR and then sacrificed and the blood, bronchoalveolar lavage fluid (BALF) and lung tissue were collected for various endpoint analysis.

### Immunohistochemistry

Lung tissues were fixed for 48 h in 4% paraformaldehyde (PFA) and embedded in paraffin. Tissue sections (5-μm) were cut and adhered to a positively charged slides. Xylene was used to deparaffinize the tissues and 100% alcohol, 95% alcohol, and normal saline (pH 7.4) was used for rehydration followed by antigen retrieval by 10 mM sodium citrate buffer, pH 6.0. Endogenous peroxidase was inactivated by 3% hydrogen peroxide following which the tissue sections were the probed with the isotype control IgG antibody or the anti- GRK2 antibody (1:200, #3982, Cell Signaling Technology, Danvers, MA) overnight at 4°C, and anti-rabbit secondary antibody (1:500, Vector Laboratories, Burlingame, CA) for 1 h at room temperature. The slides were washed and treated with the DAB substrate (Vector laboratories) for 5 min. Hematoxylin and eosin (H&E) were used as the counter stains for color development. A representative image of the isotype control antibody staining is shown in Supplemental Figure 1. Six images for each specimen were taken at 20× magnification and the images were analyzed by ImageJ software (NIH, Bethesda, MD). The determination of staining intensity for GRK2 (relative density) were performed in a blinded fashion.

### Airway Hyperresponsiveness (AHR)

Mice were anesthetized with intraperitoneal (i.p.) injection of 100 mg/kg ketamine (Henry Schein Animal Health, Dublin, OH), 10 mg/kg xylazine (Akorn, Lake Forest, IL) and 3 mg/kg acepromazine (Henry Schein Animal Health, Dublin, OH) and tracheostomized. Airway mechanics was measured using forced oscillation technique by flexiVent (SCIREQ®, Quebec, Canada). Airway resistance (Rrs) and elastance (Ers) were assessed following exposure to varying concentrations methacholine (MCh; Sigma-Aldrich, St. Louis, MO).

### Evaluation of Lung Inflammation and Goblet Cell Hyperplasia

The lungs were infused via the trachea with 10% buffered formalin, excised and stored in fresh 10% formalin overnight. Samples were then embedded in paraffin, cut into 5-μm-thick sections and stained with H&E (for lung inflammation) or Periodic acid-schiff (PAS, for goblet cell hyperplasia). Digital images of sections were obtained using a Nikon Eclipse 50i microscope (Nikon, Japan) equipped with an INFINITY-3 digital color camera (Lumenera Corporation, Canada), and INFINITY ANALYZE 6.5.4 software.

### Inflammation Scoring

Slides were scored in a blinded fashion using the scoring system described below. Six pictures covering the whole lung section at 4× magnification were collected and the total number of inflamed bronchioles (surrounded by infiltrated cells) in each picture was counted. A value from 0 to 4 was adjudged to each bronchiole scored. A value of 0–1 was decided when no inflammation or occasional cuffing with inflammatory cells was detected, a value of 2–4 for bronchi surrounded by a thin layer (2–4 cells) of inflammatory cells and a value of >4 when bronchi were surrounded by a thick layer (more than five cells) of inflammatory cells. Number of bronchioles within each category (0–1, 2–4, or >4) was then divided by the total number of inflamed bronchioles to obtain percent bronchioles within each category of inflammation. For calculating the percent severity of inflammation, the number of inflamed bronchioles that received a score of 4 or more was divided by the total number of inflamed bronchioles.

Goblet cell hyperplasia was evaluated on PAS-stained lung sections. Each lung sample was divided into 6–8 imaginary sections and digitally imaged at 10× magnification. The intensity of PAS staining in the images was assessed using ImageJ software to determine PAS positive cells as well as the percent area of PAS positive cells in each section.

### Blood Serum Collection

Blood was drawn from the superior mesenteric vein of the mouse and left at 4°C overnight. Serum was collected the next day and analyzed for total IgE and IgG1 using commercially available ELISA kits from Invitrogen (Carlsbad, CA).

### Total and Differential Leukocyte Count From Bronchoalveolar Lavage Fluid (BALF)

Cell-free BALF fluid was collected from euthanized mice after inflating the lungs with 0.5 ml of sterile PBS three times and centrifuging it at 400 × G for 5 min to separate the cellular components from the supernatants. BALF supernatants were analyzed for cytokines including, IL-4 (BD Biosciences, San Diego, CA) and IL-13 (Invitrogen) by ELISA. The BALF cellularity was determined using a hemocytometer following differential staining of the cellular fraction. Briefly, 50,000 cells were cytospun onto a clean glass slide, air- dried and then stained with Giemsa Wright stain (Sigma-Aldrich, St. Louis, MO) for 3 min. The stained slides were washed with distilled water, dried, dipped in xylene (Avantor, Radnor Township, PA) for 2 s and a cover slip was placed immediately over the cells. For each sample, a total of 200 cells were counted at 40× magnification and the number of monocytes/macrophages, lymphocytes, and eosinophils was enumerated.

### Immunomagnetic Separation of CD4 T Cells

Spleens were harvested from mice and single cell suspension was prepared. CD4 T cells were purified from the spleen cells by positive selection with the mouse naïve CD4 T cell isolation kit from Miltenyi Biotech (San Diego, CA) as per the manufacturer's recommendations. The purity of the cells as determined by flow cytometry was ≥95%.

### T Cell Stimulation *ex vivo*

CD4 T cells (2 × 10^6^ cells) were plated in a 48-well plate coated with anti-mouse CD3 antibody (BioLegend, 10 μg/ml) and anti-mouse CD28 antibody (BioLegend, 5 μg/ml) and incubated for 72 h at 37°C/5% CO_2_. The supernatant was harvested and analyzed for IL-2 by ELISA.

### Western Blotting

CD4 T cells purified from the spleen were washed twice in PBS and lysed with radio-immunoprecipitation assay (RIPA) buffer (150 mM NaCl, 1.0% Triton X-100, 0.5% sodium-deoxycholate, 0.1% sodium dodecyl sulfate, 25 mM Tris [pH 8.0], 5 mM EDTA) with protease inhibitor cocktail (Roche Applied Sciences; Mannheim, Germany). Twenty μg of protein was loaded in a 10% polyacrylamide gel for electrophoretic separation. Proteins were then transferred to nitrocellulose membranes (GE Healthcare). Membranes were blocked in 5% milk solution for 2 h, washed in Tris-buffered saline [pH 7.6] with 0.1% Tween-20 (TBST), then probed with the anti-GRK2 antibody (Cell Signaling Technology, Cat# 3982). The following day, blots were washed in TBST and probed with LiCor IRDye® 680RD or IRDye® 800CW conjugated secondary antibodies for 2 h in the dark. Blots were imaged using LiCor Odyssey Imaging Systems (Lincoln, NE) and analyzed using ImageJ software. β-Actin antibody (Cell Signaling Technology, Cat# 3700) was used as the loading control.

### Quantitative Real-Time PCR

RNA was isolated from mouse lungs using the TRIzol reagent (Invitrogen) and was reverse transcribed into cDNA using the High-Capacity cDNA reverse transcription kit (Applied Biosystems, Foster City, CA). Real-time quantitative PCR was performed using Quant Studio™ 3 system (Applied Biosystems) with validated Taqman primers and Fast Advanced Master Mix. Relative gene expression data (fold change) between samples was accomplished using the 2^−ΔΔCt^ method. GAPDH was used as the internal control.

### *In vitro* HDME Re-stimulation

Lungs were harvested from mice subjected to the asthma model and single cells were isolated using the Percoll density method. Briefly, lung samples were minced and digested with collagenase P (1 mg/ml, Roche Diagnostics, Indianapolis, IN) at 37°C for 30 min. Single cell suspension was obtained by passing the digested tissue through a 70-μm cell strainer (Alkali Scientific Inc., Fort Lauderdale, FL) with a plunger. Lung mononuclear cells were then isolated using density centrifugation with 70% Percoll (GE, Piscataway, NJ). The isolated cells were washed and resuspended in RPMI media (Life Technologies, Carlsbad, CA) conditioned with 10% FBS (Atlanta Biologicals, Flowery Branch, GA) and 1% penicillin-streptomycin (Mediatech Inc., Manassas, VA). The cells (2 × 10^5^ cells/well/200 μL) were plated in a 96 well plate and stimulated with HDME (3 μg/well). After 72 h the supernatant was harvested and was analyzed for cytokines (IL-4 and IL-13) by ELISA.

### Statistics

Statistical significance was determined using GraphPad Prism software (GraphPad, San Diego, CA). Student's unpaired *t*-test assuming equal standard deviation or two-way ANOVA was used for analysis of data. Significance is shown as ^*^p < 0.05 and ^**^p < 0.01.

## Results

### GRK2 Expression Is Enhanced in the Lungs of Human Asthmatic Patients and Mice Exposed to HDME

**Figure 1:**
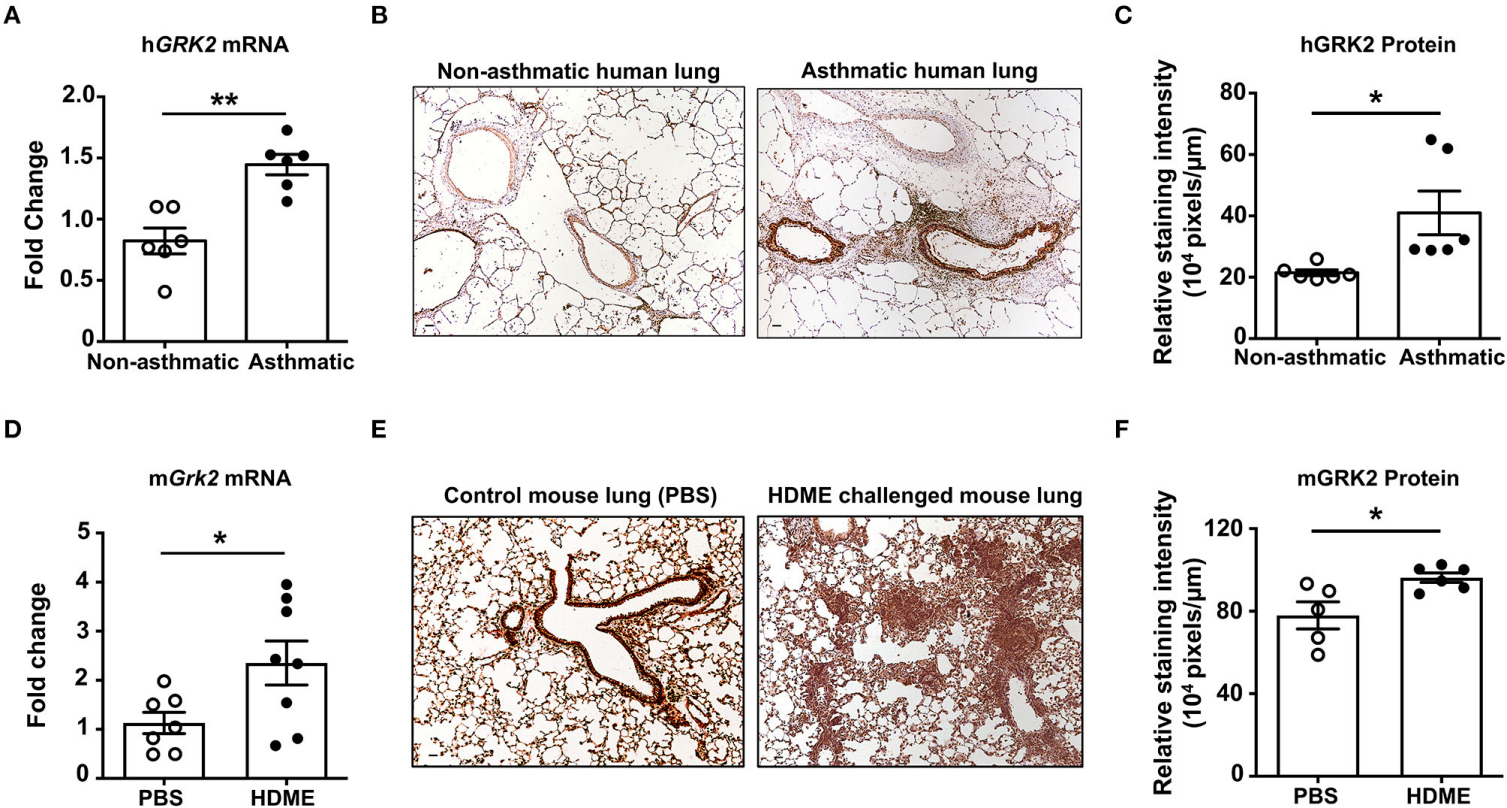
Increased levels of GRK2 in the lungs of human asthmatics and HDME-challenged mice. **(A)** Whole lung tissue from healthy donors and asthma patients were analyzed for mRNA expression of human (h)GRK2. Data is shown as mean fold change ± SEM with a total of 6 samples per cohort. **(B,C)** Immunohistochemistry (IHC) was performed to determine GRK2 levels in the lungs of healthy donors and asthma patients. **(B)** Representative images from 6 donors for each group and **(C)** relative staining intensity of GRK2 expression are shown. **(D–F)** Male and female B6 mice were challenged intranasally (i.n.) with PBS or HDME (50 μg) on alternate days for a total of seven injections. Twenty-four hours after the last treatment mice were euthanized and the lung tissue was harvested and **(D)** analyzed for mRNA expression of mouse (m)GRK2. Data is shown as mean fold change ± SEM from 7 to 8 samples per cohort. IHC of mouse lung tissues were performed and **(E)** representative images of GRK2 staining in the lungs of 5–6 mice from each cohort is shown. **(F)** Relative staining intensity of GRK2 expression in the mouse lung samples is shown. Scale bar = 100 μm. Statistical significance was determined by using Student's unpaired *t* test. *p ≤ 0.05, **p ≤ 0.01.

GRK2 is a ubiquitously expressed adapter protein that is present in most cell types of the airways including AECs, ASMCs and other immune cells. Its expression however is altered during pathological conditions; for e.g., the expression levels of GRK2 is enhanced in cystic fibrosis patients (25). To determine if GRK2 is similarly upregulated in the lungs during asthma, we performed qRT-PCR and IHC staining on lung tissue sections from deceased asthmatic and non-asthmatic (control) individuals. GRK2 expression (both at the mRNA and protein levels) was significantly elevated in the lungs of asthmatic individuals (Figures 1A–C). To test if GRK2 levels are affected in a mouse model of human asthma, we treated wild-type B6 mice with HDME on alternative days (for a total of seven exposures) and harvested the lung tissue 24 h following the final exposure. Consistent with our data from human lung samples, GRK2 expression (mRNA and protein) was increased in the lung tissues of mice exposed to HDME as compared to PBS controls (Figures 1D–F).

### GRK2 Promotes Th2 Cytokine Production but Not AHR, Serum IgE Levels and Lung Immune Cell Infiltration in HDME-Treated Mice

**Figure 2:**
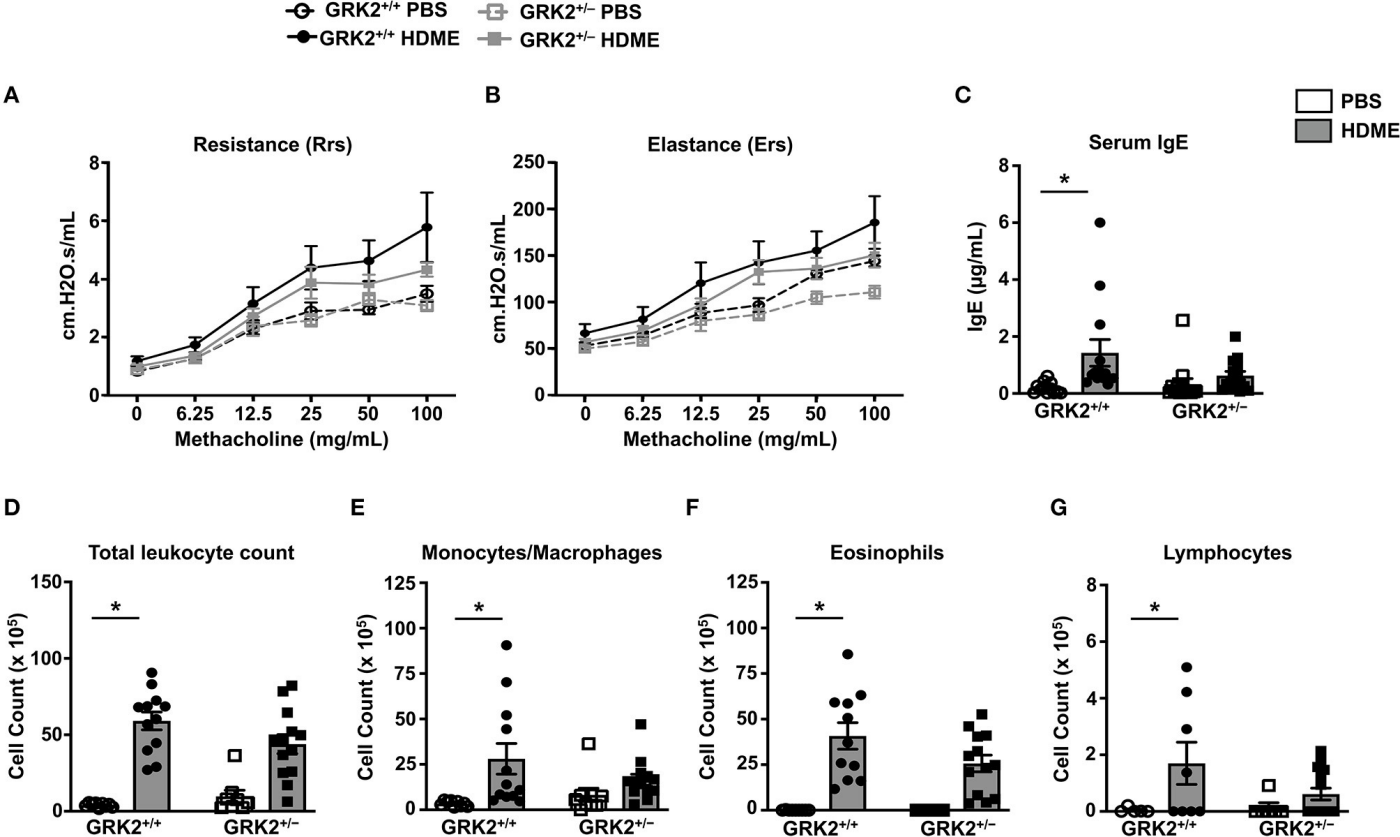
AHR, serum IgE and immune cell counts in the BALF are not altered in GRK2^+/−^ mice challenged with HDME. **(A,B)** PBS control or HDME-exposed GRK2^+/+^ and GRK2^+/−^ mice were anesthetized 24 h after the last HDME challenge and analyzed for AHR. **(A)** Lung constriction [Resistance [Rrs]] and **(B)** elastic stiffness of lungs, chest walls, and airways [Elastance [Ers]] after challenge with increasing doses of methacholine is shown. **(C)** Levels of serum IgE in GRK2^+/+^ and GRK2^+/−^ mice treated with PBS or challenged with HMDE. **(D)** Total leukocyte counts and differential cell counts of **(E)** monocytes/macrophages, **(F)** eosinophils, and **(G)** lymphocytes are shown. Data are presented as mean ± SEM and are pooled from 2 to 3 independent experiments with a total of 5–14 mice per cohort. Both male and female mice were used for these experiments. *p < 0.05.

Because GRK2 expression was elevated in the lungs of HDME-challenged mice, we hypothesized that it would regulate the asthmatic response in a mouse model. To test this, we used GRK2^+/−^ mice (GRK2^−/−^ is embryonically lethal) and GRK2^+/+^ (control) mice and exposed them to PBS or HDME. AHR parameters such as central airway resistance (Rrs) and lung elastance (Ers) were assessed in response to varying concentrations of Mch (6.25–100 mg/ml). There was no difference in the baseline Rrs and Ers were between GRK2^+/+^ and GRK2^+/−^ mice injected with PBS (Figures 2A,B). HDME-exposed GRK2^+/+^ and GRK2^+/−^ mice demonstrated elevation in Rrs and Ers measurements in a dose-dependent manner to Mch. However, there was no difference in HDME-induced Rrs and Ers values between the two cohorts of mice. Allergic asthma is characterized by elevated levels of serum IgE. We observed that serum IgE was higher in HDME-treated GRK2^+/+^ mice as compared to PBS controls (Figure 2C). Although, GRK2^+/−^ mice had lower serum IgE as compared to HDME exposed GRK2^+/+^ mice, the values were not significantly different.

Airway inflammation is accompanied by an influx of inflammatory cells such as monocytes/macrophages, eosinophils and lymphocytes into the lungs and these cells are consistently detected in the BALF of allergic individuals. Since GRK2 has been previously reported to affect migration of immune cells (23, 26–28), we next determined the cellularity in BALF of GRK2^+/+^ and GRK2^+/−^ mice. Following exposure to HDME, an increase in immune cell numbers was seen in the BALF of GRK2^+/+^ mice. The inflammatory infiltrate mainly consisted of eosinophils along with lower numbers of monocytes/macrophages and lymphocytes. Surprisingly, there was no significant difference in the total leukocyte, monocyte/macrophage, eosinophil or lymphocyte counts between HDME treated GRK2^+/−^ and GRK2^+/+^ mice (Figures 2D–G).

**Figure 3:**
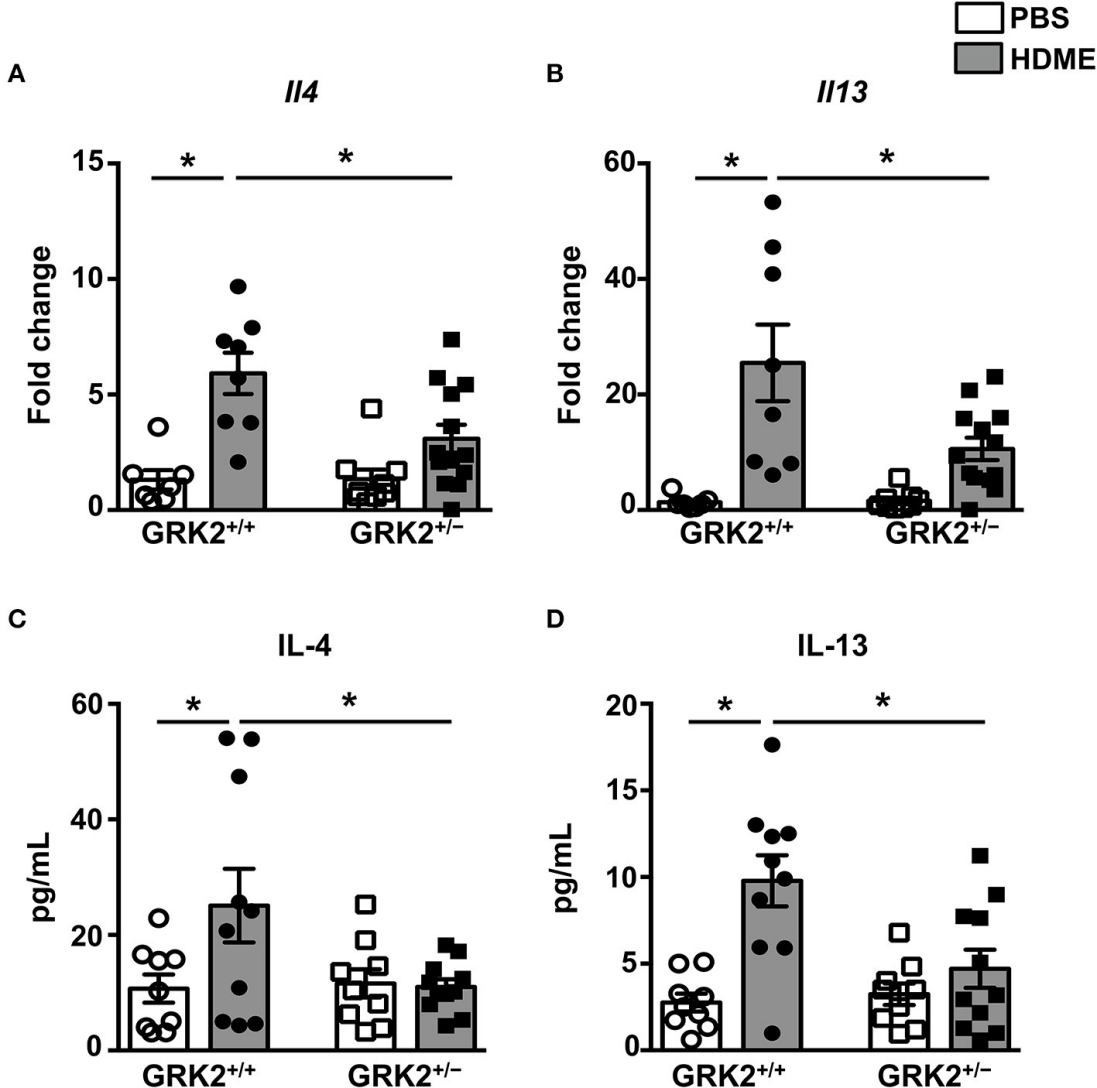
IL-4 and IL-13 levels in the lungs and BALF are reduced in GRK2^+/−^ mice exposed to HDME. PBS control or HDME-exposed GRK2^+/+^ and GRK2^+/−^ mice were euthanized after the last HDME challenge. **(A,B)** Lungs samples from these mice were analyzed for mRNA expression of Th2 cytokines [**(A)** *Il4* and **(B)** *Il13*]. **(C,D)** BALF was harvested from these mice and **(C)** IL-4 and **(D)** IL-13 cytokine levels were determined. Data are presented as mean ± SEM and are pooled from 2 to 3 independent experiments with a total of 7–13 male and female mice per cohort. Statistical significance was determined Student's unpaired *t*-test. *p ≤ 0.05.

A previous study from our laboratory showed that repeated HDME treatment results in increased levels of Th2 cytokines in the lungs (29), so we next analyzed Th2 cytokines in the lung tissues and BALF of different cohorts of mice (Figure 3). As expected the mRNA (Figures 3A,B) and protein levels of IL-4, and IL-13 (Figures 3C,D) were elevated in the lungs and BALF of HDME-exposed GRK2^+/+^ mice as compared to PBS controls. Interestingly, the expression levels of these cytokines were significantly reduced in GRK2^+/−^ HDME mice (Figures 3A–D).

### GRK2 Expression Is Required in T Cells for HDME-Induced AHR and Serum IgE Production

**Figure 4:**
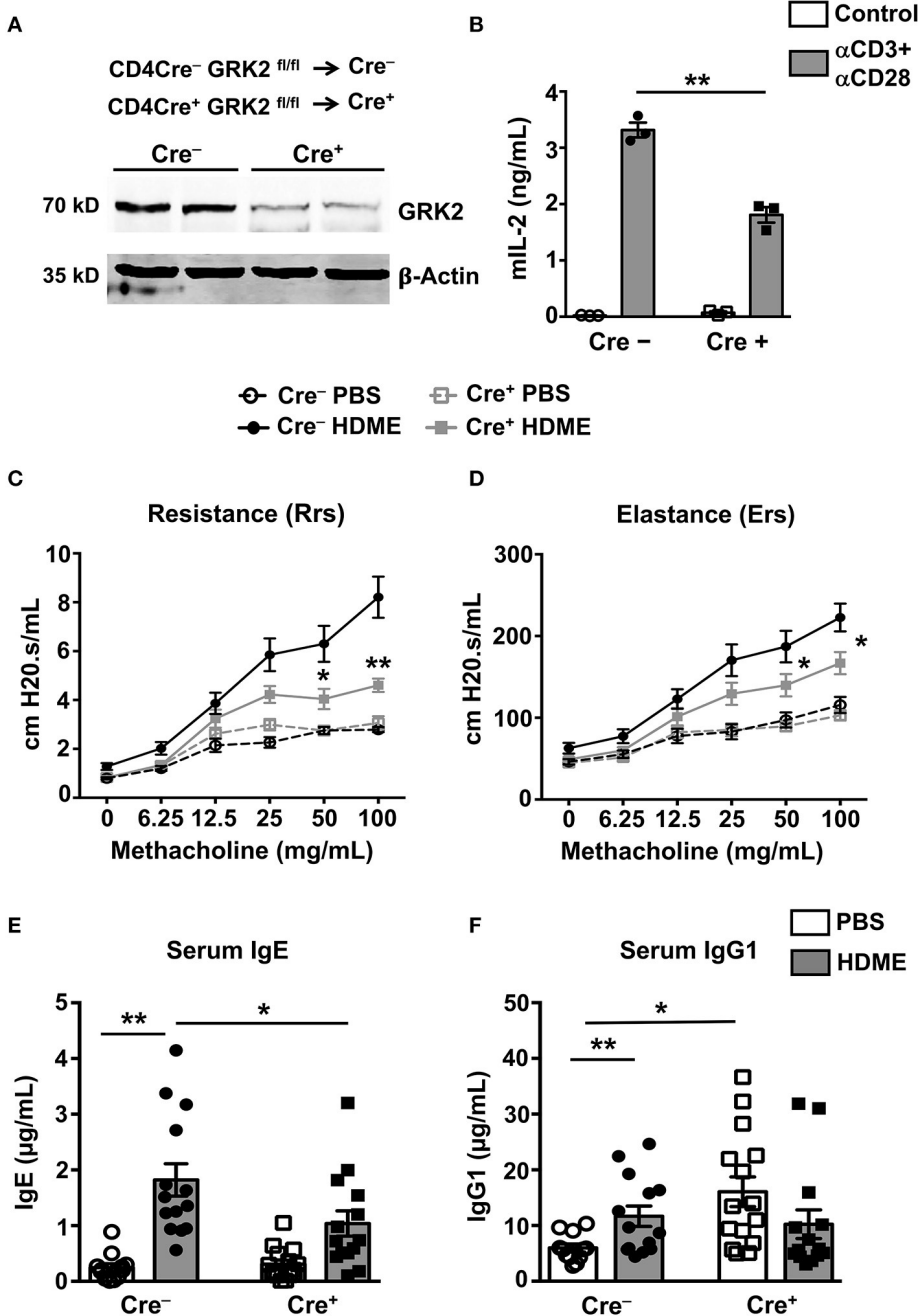
Mice lacking GRK2 specifically in T cells show attenuated AHR and serum IgE levels following HDME challenge. **(A)** CD4 T cells were purified from splenocytes of Cre^−^ and Cre^+^ mice and western blotting was performed to determine GRK2 levels in CD4 T cells. A representative blot with samples from two mice per group is shown. **(B)** CD4 T cells isolated from Cre^−^ and Cre^+^ mice were stimulated with plate bound anti-CD3 (10 μg/ml) and anti-CD28 antibodies (5 μg/ml) for 72 h and the supernatants were analyzed for IL-2 production by ELISA. **(C,D)** PBS control or HDME-exposed Cre^−^ and Cre^+^ mice were anesthetized 24 h after the last HDME challenge and analyzed for AHR. **(C)** Rrs and **(D)** Ers values after challenge with increasing doses of methacholine are shown. Statistical significance for the AHR experiments was determined by two-way ANNOVA with Bonferroni's correction. *p ≤ 0.05, **p ≤ 0.01. **(E,F)** Bar graphs show levels of **(E)** serum IgE and **(F)** serum IgG1 in Cre^−^ and Cre^+^ mice treated with PBS or challenged with HMDE. Data are presented as mean ± SEM and are pooled from 2 to 3 independent experiments with a total of 6–15 mice per cohort. Both male and female mice were used for the HDME experiments. Statistical significance was determined Student's unpaired *t*-test. *p ≤ 0.05, **p ≤ 0.01.

Given that the Th2 cytokines (IL-4 and IL-13) are reduced in the lungs of GRK2^+/−^ mice, it is possible that GRK2 modulates T cell responses during asthma. To determine the role of T cell-specific GRK2 in affecting the asthma pathology, we generated a mouse stain [CD4 Cre^+^ GRK2^fl/fl^ (Cre^+^)] that lacks GRK2 in T cells. We then used the Cre^+^ and littermate CD4 Cre^−^ GRK2^fl/fl^ (Cre^−^) mice (as controls) for our experiments. First, we purified CD4 T cells from the spleens of these mice and analyzed for GRK2 expression in these cells using Western Blotting. As shown in Figure 4A, GRK2 expression was reduced by ~80% in CD4 T cells from Cre^+^ mice. A previous report had demonstrated that GRK2 promotes T cell activation and is required for production of cytokines such as IL-2 (24). To test this in our model, we stimulated T cells isolated from the Cre^+^ and Cre^−^ with anti-CD3 and anti-CD28 antibodies and assessed for IL-2 secretion as a measure of T cell activation. Our data suggests that IL-2 production was significantly reduced in Cre^+^ cells that had reduced levels of GRK2 (Figure 4B). We then subjected the Cre^−^ and Cre^+^ mice to the HDME asthma model, performed AHR experiments and determined serum IgE and IgG1 levels in allergic mice. In contrast to the results obtained with GRK2^+/−^ mice, both the AHR (Rrs and Ers values) (Figures 4C,D) and serum IgE levels (Figure 4E) were significantly reduced in Cre^+^ mice when compared to Cre^−^ mice following HDME exposure. This suggests that GRK2 is required in the T cell compartment to modulate AHR and serum IgE levels in our asthma model. Interestingly, though IgG1 was unaltered between the Cre^+^ and Cre^−^ mice treated with HDME, Cre^+^ mice had higher basal levels of this antibody as compared to Cre^−^ mice (Figure 4F).

### T Cell-Specific GRK2 Expression Promotes Lung Inflammation and Th2 Cytokine Production in an HDME-Induced Allergic Asthma Model

**Figure 5:**
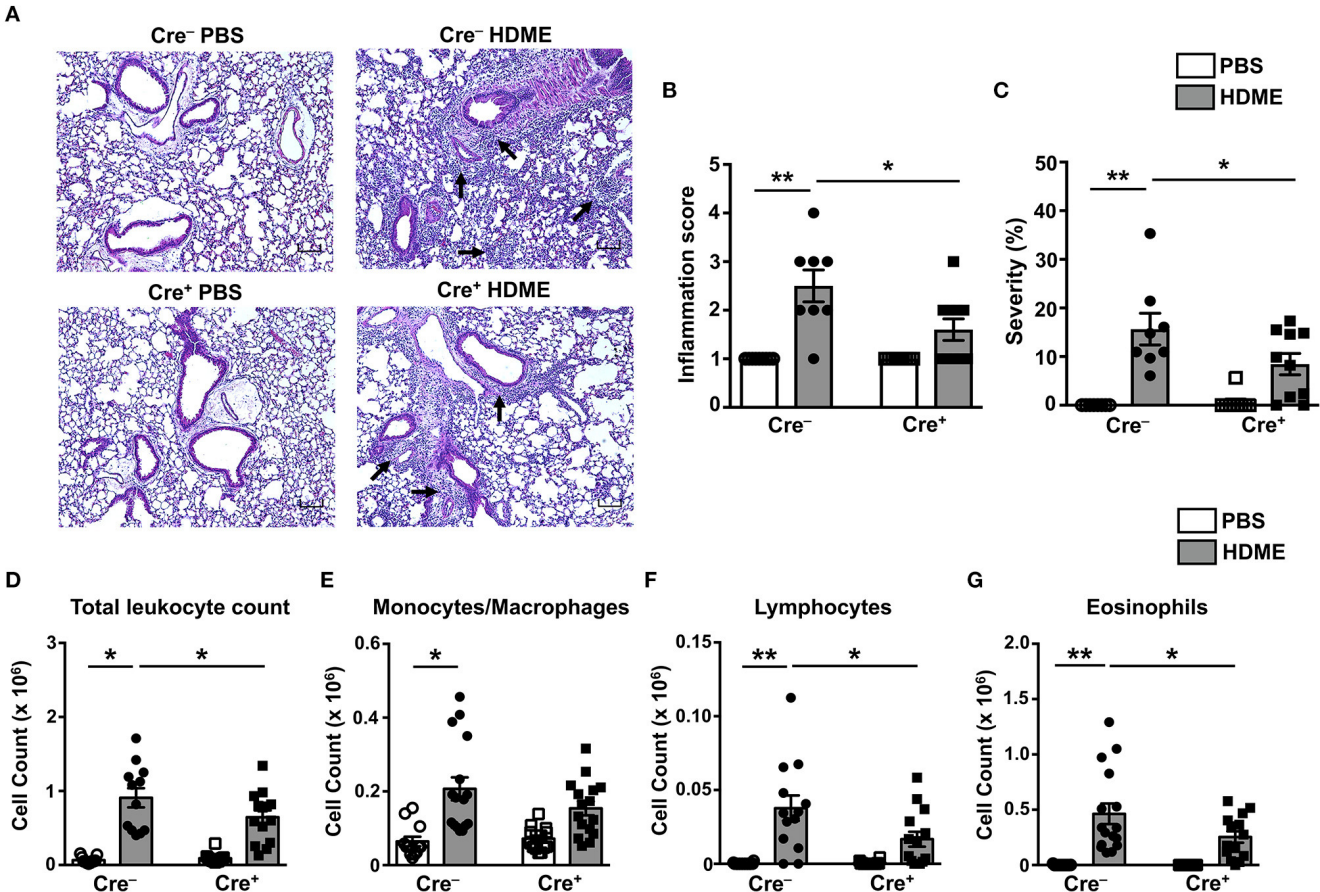
Reduced airway inflammation and immune cell infiltration in mice lacking GRK2 in T cells. **(A)** Hematoxylin and eosin (H&E) staining of lung section of Cre^−^ and Cre^+^ mice injected with PBS or HDME. Scale bar = 100 μm. Representative images of the lungs at 4× magnification is shown. Black arrows indicate cellular infiltration (inflammation) around the bronchioles and blood vessels. **(B)** Bronchial inflammation and **(C)** severity scores in PBS and HDME-treated Cre^−^ and Cre^+^ mice are shown. **(D–G)** BALF from Cre^−^ and Cre^+^ mice injected with PBS or challenged with HDME were analyzed for **(D)** total leukocyte counts and differential cell counts of **(E)** monocytes/macrophages, **(F)** lymphocytes and **(G)** eosinophils. Data are presented as mean ± SEM and are pooled from three independent experiments with a total of *n* = 5–7 for PBS and *n* = 13–16 mice for HDME-treated groups. Both male and female mice were used for this experiment Statistical significance was determined by Student's unpaired *t*-test. *p ≤ 0.05, **p ≤ 0.01.

To determine if GRK2 expression in T cells regulates airway inflammation, we harvested the lungs from Cre^+^ and Cre^−^ mice exposed to HDME and performed H&E staining on these tissues. We also enumerated the numbers of monocytes/macrophages, eosinophils and lymphocytes in the BALF. HDME treatment resulted in a significant increase in airway inflammation in Cre^−^ mice as shown by the intense H&E staining especially near the peri-bronchial and perivascular areas of the lung tissue. However, this inflammation was severely attenuated in the Cre^+^ mice exposed to HDME (Figures 5A–C). In addition, Cre^+^ HDME mice exhibited reduced total BALF cell counts (Figure 5D). Specifically, while there was no change in monocyte/macrophage numbers (Figure 5E), eosinophil and lymphocyte counts were significantly lower in the BALF of Cre^+^ HDME mice (Figures 5F,G).

**Figure 6:**
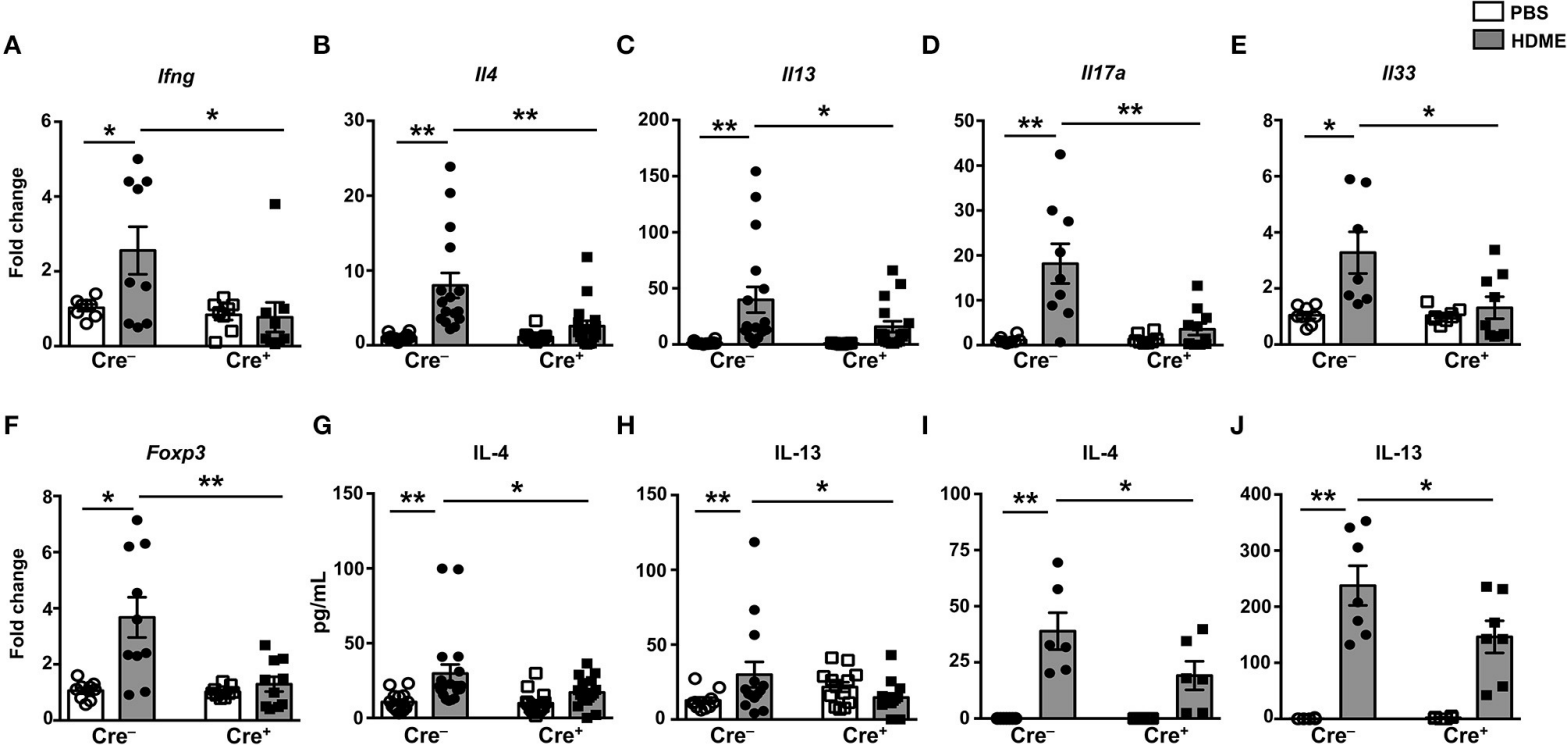
Mice with GRK2-deficient T cells have reduced inflammatory cytokines in lungs following exposure to HDME. **(A–F)** Lungs from Cre^−^ and Cre^+^ mice injected with PBS or HDME were analyzed for gene expression of **(A)** the Th1 cytokine (*Ifng*), **(B,C)** Th2 cytokines (*Il4* and *Il13*), **(D)** Th17 cytokine (*Il17a*), **(E)** alarmin (*Il33*), and **(F)** Foxp3 (*Foxp3*). **(G)** IL-4 and **(H)** IL-13 cytokine levels in the BALF supernatant of Cre^−^ and Cre^+^ mice challenged or not with HMDE are shown. **(I,J)** Lung mononuclear cells were harvested from Cre^−^ and Cre^+^ mice injected with PBS or HDME. The cells were re-exposed to HDME and **(I)** IL-4 and **(J)** IL-13 cytokine levels in the supernatant were estimated. Data is shown as mean ± SEM and pooled from 2 to 3 independent experiments with a total of 6–17 male and female mice per cohort Statistical significance was determined by Student's unpaired *t*-test. *p ≤ 0.05, **p ≤ 0.01.

It is well established that the Th1-Th2 axis is skewed during asthma pathogenesis and that the Th1 cytokines can modulate the expression levels of Th2 cytokines and vice-versa (30, 31). To determine if T cell-specific GRK2 expression modulates Th1 and Th2 cytokines, we estimated the mRNA levels of Th1 (*Ifng*) and Th2 (*Il4* and *Il13*) cytokines in lungs of mice exposed to HDME. We observed a significant reduction in *Ifng* in the Cre^+^ mice as compared to the control Cre^−^ mice (Figure 6A). Consistent with the results obtained with GRK2^+/−^ mice (Figure 3), and reduced inflammation observed in the Cre^+^ HDME mice (Figure 5), *Il4* and *Il13* levels were significantly reduced in the lungs of Cre^+^ HDME mice (Figures 6B,C). Recent reports have suggested that the cytokines of the IL-17 family are increased in BALF samples from patients with asthma and IL-17A has been predicted to be a risk factor for severe asthma (32, 33). Accordingly, we observed higher levels of *Il17a* in the lungs of HDME-exposed Cre^−^ mice (Figure 6D). However, this was reduced in the Cre^+^ mice. Furthermore, epithelial cell-derived cytokines such as IL-33 play a major role in the initiation of allergic asthma (34). We observed an increase in *Il33* levels in HDME-treated Cre^−^ mice that was significantly attenuated in Cre^+^ HDME mice (Figure 6E). Another important immune cell type that has been implicated in the pathology of asthma are regulatory T cells (Tregs) that express the transcription factor Foxp3. Specifically, several reports have shown that functional defects in Tregs leads to enhancement of Th2 responses and the pathogenesis of asthma (35–38). We observed an increase in *Foxp3* expression following HDME exposure in Cre^−^ mice that was significantly decreased in Cre^+^ mice (Figure 6F). Consistent with the mRNA expression data (Figures 6B,C), we also observed a reduction in both IL-4 and IL-13 protein levels in the BALF of Cre^+^ HDME mice as compared to allergen-challenged Cre^−^ mice (Figures 6G,H). Additionally, this defect in IL-4 and IL-13 cytokine production was also observed when lung mononuclear cells from HDME-treated Cre^+^ mice were re-exposed to the allergen *in vitro* (Figures 6I,J).

### HDME-Induced Gene Expression of Polymeric Mucins and Goblet Cell Hyperplasia Is Reduced in Mice That Lack GRK2 in T Cells

**Figure 7:**
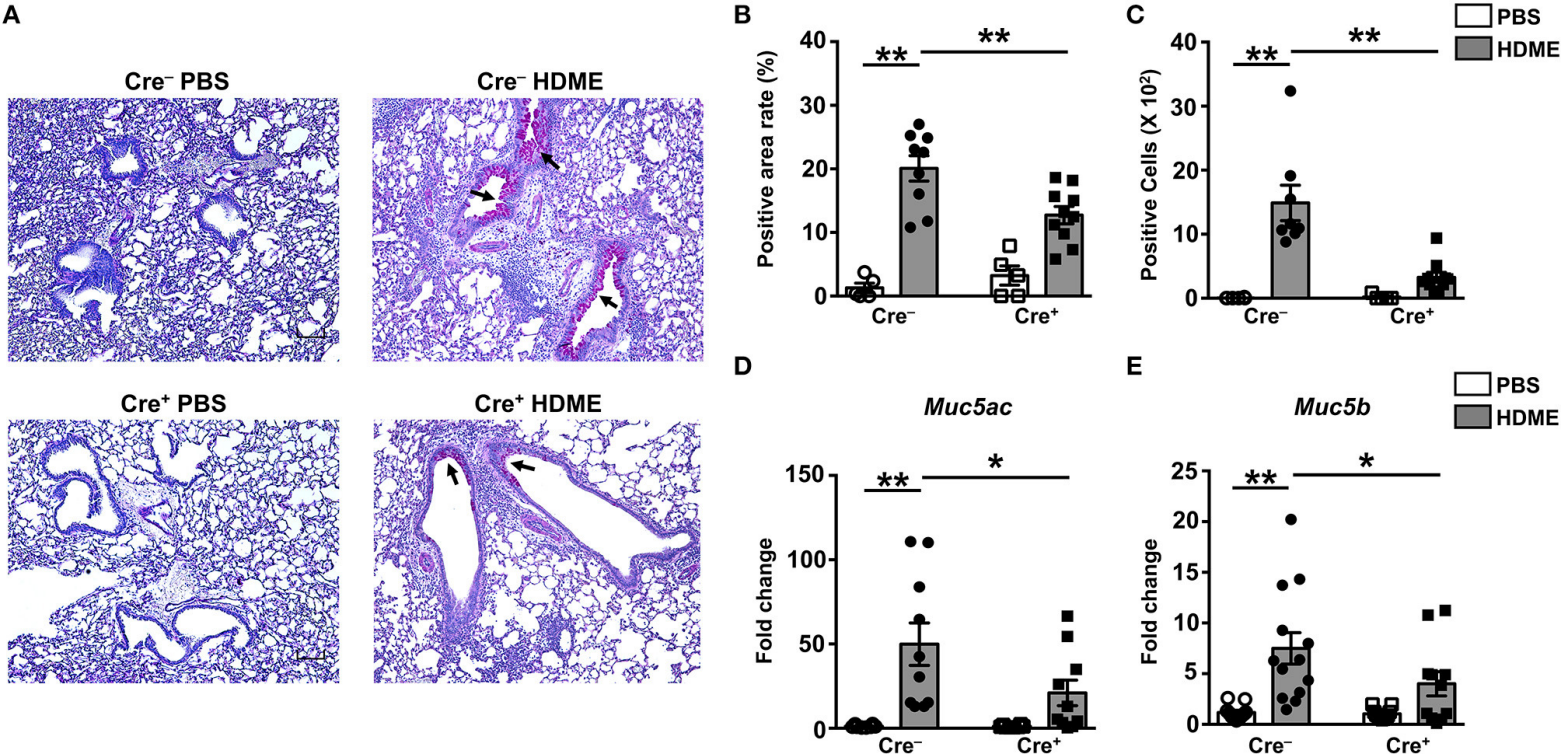
Decreased goblet cell hyperplasia and mucin production in the lungs of HDME-challenged mice that lack GRK2 in T cells. Cre^−^ and Cre^+^ mice were challenged with PBS or HDME. Twenty-four hours after the last injection of HDME, mice were sacrificed, and lung tissues were analyzed for **(A–C)** PAS staining and **(D,E)** mRNA expression of polymeric mucins [**(D)** *Muc5ac* and **(E)** *Muc5b*]. Representative lung images [**(A)**, 4× magnification, arrows indicate PAS staining] and **(B)** % PAS positive area and **(C)** PAS positive cell counts are shown. Scale bar = 100 μm. Data in **(B–E)** are presented as mean ± SEM and are pooled from 2 to 3 independent experiments with a total of 5–14 male and female mice per cohort. Statistical significance was determined using Student's unpaired *t*-test. *p ≤ 0.05, **p ≤ 0.01.

Goblet cell hyperplasia (an increase in the numbers of goblet cells) is a characteristic feature of allergic asthma that results in excessive mucus production and airflow obstruction in the lungs. Increased level of Th2 cytokines such as IL-13 has been frequently associated with mucus secretion and is consequently believed to be a major contributor of goblet cell hyperplasia during asthma (39). We analyzed goblet cell hyperplasia through PAS staining of lung sections from Cre^−^ and Cre^+^ mice. Consistent with the diminished IL-13 levels (Figures 6C,H), HDME-treated Cre^+^ mice exhibited reduced PAS staining (Figure 7A). We also quantified percent area of PAS+ staining and PAS+ cell count and observed a significant decrease in these parameters in the HDME-challenged Cre^+^ mice (Figures 7B,C). The polymeric mucins, Muc5ac and Muc5b are primarily responsible for mucus production in mouse models of asthma (40, 41). We observed that the mRNA levels of both *Muc5ac* and *Muc5b* were significantly upregulated in the lungs of Cre^−^ HDME-exposed mice (Figures 7D,E), however, they were significantly decreased in Cre^+^ mice.

## Discussion

Allergic asthma is a chronic airway inflammatory disease that affects ~300 million people worldwide, and its prevalence has increased considerably in recent years. It is now realized that the pathogenesis of asthma is complex and involves multiple cells types such as the AECs, ASMCs, immune cells and cells of the nervous system. GRK2 is a ubiquitously expressed serine/threonine kinase that has been previously reported to control the activities of different cell types associated with the asthma pathology. Specifically, GRK2 inhibits β_2_AR signaling in the AECs and ASMCs to alter the contractility of the airways (42). GRK2 attenuates T cell chemotaxis and regulates their cytokine production following activation (23, 24). We have previously shown that GRK2 regulated mast cell responses to the C3a (43) and the IgE receptors (14), which play an important role in mediating allergic asthma in humans (44, 45). While all of these previous studies implicate a role for GRK2 in possibly regulating the asthma pathology, our current study is the first to test this contention. Herein, we show that GRK2 regulates the production of Th2 cytokines (IL-4 and IL-13) in the lungs of mice exposed to HDME, a physiologically relevant allergen. Importantly, GRK2 expression within T cells is required for the manifestation of characteristic features of asthma such as AHR, airway inflammation, IgE and Th2 cytokine (IL-4 and IL-13) production, goblet cell hyperplasia and mucus secretion.

GRK2 levels are increased in different tissues of patients and in preclinical models of cardiovascular diseases and contribute to disease progression. Specifically, enhanced GRK2 expression has been reported in the failing human hearts (46) and in experimental models of heart failure, in both chronic hypertensive, and ischemic disease (7). GRK2 levels in the brain is increased during early stages of damage in Alzheimer's disease patients and consequently, GRK2 was predicted to be a marker for early hypoperfusion-induced brain damage, which is associated with mitochondrial damage found in Alzheimer's disease patients (47). In addition, GRK2 is upregulated in the lungs of the cystic fibrosis patients and this correlated with decreased β_2_AR expression and activity in these individuals (25). In agreement with these reports, we observed enhanced GRK2 levels in the lung sections from human asthma patients and mice that were treated with HDME. The enhancement in GRK2 levels is possibly due to: (1) accumulation of immune cells, (2) proliferation of ASMCs, and/or (3) increased GRK2 expression in different cell types in the lungs. While we did observe an increase GRK2 staining in the human and mouse lung tissues by IHC, it was difficult to determine if GRK2 was upregulated in any particular cell type. Additionally, it remains to be determined if the increase in GRK2 expression is accompanied with enhanced GRK2 activity in the lungs during asthma.

Our experiments with GRK2^+/−^ mice revealed a selective reduction of IL-4 and IL-13 cytokines in the lungs but no effect on AHR and immune cell counts. These results suggest that GRK2 has differential effects on various cell types (AECs, ASMCs, and several immune cells) present in the lungs. Also, the partial expression of GRK2 in the GRK2^+/−^ mice may account for the moderate phenotype observed in these animals. Regardless, we observed a significant reduction in AHR and all parameters of airway inflammation including decreased leukocyte counts and reduced levels of IL-4 and IL-13 in the BALF of mice that lacked GRK2 in T cells (Cre^+^ mice). Both IL-4 and IL-13 cause immunoglobulin class switch to IgE during asthma. In agreement with this, IgE levels were reduced in the serum of Cre^+^ mice. IL-4 and IL-13 production by lung mononuclear cells obtained from Cre^+^ mice following re-exposure to HDME was also attenuated. CD4 T cells purified from splenocytes of Cre^+^ mice showed attenuated IL-2 production following stimulation of TCR via CD3 and CD28 ligation *in vitro*. All these data suggest that GRK2 regulates Th2 cytokine production by T cells *in vitro*. Our results are is in line with previous reports from our laboratory and others that have demonstrated that this adapter molecule regulates cytokine (IL-6 and IL-13) production in mast cells (14) and T cells (24).

It is well established that GRK2 is a critical regulator of GPCR signaling and since chemokine receptors are GPCRs, GRK2 has been previously shown regulate migration of cells. Accordingly, GRK2 inhibits T cell chemotaxis to CCL3, CCL4, and CCL5 which act through the chemokine receptors CCR1 and CCR5 (23). These chemokines and their receptors have been implicated in exacerbating airway inflammation and are chemotactic for T cells (48). It was therefore surprising that we observed a reduction in lymphocyte counts in the BALF of Cre^+^ mice as compared to the control Cre^−^ mice, given that GRK2 negatively regulates chemotaxis of T cells. A recent report showed that IL-4 promotes migration of Th2 cells into the lungs (49). Since we observed a reduction in IL-4 in the lungs of Cre^+^ mice exposed to HDME, it is conceivable that that reduced lymphocyte counts in the lungs is a result of reduced migration of these cells due to decreased IL-4 levels. Additionally, it is also possible that GRK2 directly regulates the chemotaxis by acting as an adapter molecule. For example, GRK2 can stimulate chemotactic migration of epithelial cells by modulating the formation and/or disassembly of integrin-based cell-extracellular matrix contacts (50). GRK2 also serves as a RhoA-activated scaffold protein for mitogen activated protein kinase activation and promotes cell migration (50, 51). Whether GRK2 regulates these or other mechanisms in T cells to regulate leukocyte infiltration into lungs remains to be determined.

IL-13 plays a pivotal role in regulating mucus production during asthma. Specifically blockade of IL-13 using a soluble antibody resulted in attenuation of mucus production while intranasal administration of this cytokine resulted in exacerbation of this response (52, 53). Also, transgenic mice overexpressing IL-13 have increased mucus production (54) whereas *Il13*^−/−^ mice have a marked reduction in mucus secretion (55). Herein, we demonstrate that gene expression of the polymeric mucins, Muc5ac and Muc5b, mucus production and goblet cell hyperplasia are significantly reduced in the lungs of allergen-treated Cre^+^ that have reduced expression of GRK2 in T cells. IL-13 signal induces Muc5ac expression in asthma (56). Muc5b is upregulated in human bronchial epithelial cell stimulated with IL-13 (57). Also, IL-13 has been shown to directly increase goblet cell formation producing Muc5b in primary human airway basal cell culture in Matrigel (58). Thus, it is likely that the reduction in IL-13 levels observed in Cre^+^ mice may contribute to the attenuated mucus production and goblet cell hyperplasia observed in these animals.

An interesting finding of the current study is that the alarmin *Il33* is reduced in the lungs of the Cre^+^ mice exposed to HDME. AECs produce IL-33 following allergen exposure. IL-33 promotes Th2-associated immune responses via maturation and activation of effector Th2 cells, innate lymphoid cells (ILCs) and other innate immune cells such as eosinophils (59). Accordingly, we observed a reduction in eosinophil numbers in the BALF of HDME treated Cre^+^ mice. Recent evidence indicates that cytokines of the IL-17 family are also increased in BALF samples from patients with asthma. Specifically, IL-17A levels are upregulated in these individuals and IL-17 polymorphisms is frequently associated with asthma (32, 33). We observed a significant reduction in *Il17a* mRNA levels in HDME-exposed Cre^+^ mice that correlated with attenuated airway inflammation observed in these animals. Collectively, GRK2 in T cells regulates IL-33 and IL-17A in the lungs of mice challenged with HDME. The mechanisms that regulate the levels of these cytokines are complex. Other cytokines such as IL-27 (60) and cell types such as mast cells (61) and ILC2s' (62) are involved in pathways that modulate IL-17A and IL-33. Additionally, IL-33 promotes IL-13 production (63) and is also a chemoattractant for Th2 cells (64). Thus, it is possible that the lower IL-13 levels and reduced BALF lymphocyte counts observed in mice that lack GRK2 in T cells is because of reduced IL-33 in the lungs. Previous reports have showed that Tregs attenuate Th2 responses in allergic asthma (35–38). However, we observed that the Foxp3 mRNA levels were reduced in the lungs of HDME exposed Cre^+^ mice suggesting the presence of lower numbers of Tregs. It is possible that a reduction in GRK2 results in decreased migration of Tregs to the lungs. It is currently unclear how GRK2 in CD4 T cells regulates cytokine production and Foxp3 expression and/or Treg numbers in the lungs of mice exposed to HDME.

The exact mechanism by which GRK2 regulates T cell activation, that in turn, controls HDME-induced allergic response is currently unknown. In a recent report Dinkel et al. (24) showed that GRK2 is required for the TCR-mediated transactivation of the chemokine receptor CXCR4 and mediates phosphorylation of the CXCR4 which is required for TCR-CXCR4 complex formation. Additionally, depletion or pharmacologic inhibition of GRK2 attenuated the ability of T cells to produce IL-2 and IL-10. In the same study the authors also propose that TCR/CD3 stimulation induces tyrosine phosphorylation of GRK2 via a mechanism requiring a Src kinase. Prior studies have demonstrated that GRK2 can physically associate with the TCR (65) and phosphorylation of GRK2 at Tyr-13, Tyr-86, and Tyr-92 by Src kinases enhances its kinase activity (66). Thus, TCR-mediated activation of Src kinases could lead to phosphorylation of GRK2 that can affect T cell response. It is also possible that GRK2 affects T cell activation via other mechanisms. We have previously demonstrated that GRK2 promotes mast cell activation and cytokine production independent of its kinase activity (14). Specifically, GRK2 modulated the activation of the kinase Syk and the regulator of G protein signaling homology (RH) domain of GRK2 was required for its functions in mast cells (14). In summary, our study provides evidence that a reduction in GRK2 levels in T cells attenuates airway inflammation, mucus production, AHR, Th2 (IL-4 and IL-13) cytokines and serum IgE levels in allergen-treated mice. Further studies clarifying the mechanism through which GRK2 regulates T cell responses will help usher in a new approach for clinical management of asthma symptoms in patients that are resistant to corticosteroid therapy.

## Data Availability Statement

The original contributions presented in the study are included in the article/Supplementary Material, further inquiries can be directed to the corresponding author/s.

## Ethics Statement

The studies involving human participants were reviewed and approved by Institutional Review Board at Rutgers University. Written informed consent for participation was not required for this study in accordance with the national legislation and the institutional requirements. The animal study was reviewed and approved by Institutional Animal Care and Use Committee at Michigan State University.

## Author Contributions

AK performed experiments and analyzed data. CY performed experiments. RP provided lung samples and edited the manuscript. RD planned experiments, analyzed data, and wrote parts of the manuscript. HS conceived the study, designed experiments, analyzed data, and wrote the manuscript. All authors contributed to the article and approved the submitted version.

## Conflict of Interest

The authors declare that the research was conducted in the absence of any commercial or financial relationships that could be construed as a potential conflict of interest.
