# Supplementary material for: G Protein-Coupled Receptor Kinase 2 (GRK2) Regulates T Cell Response in a Murine Model of House Dust Mite-Induced Asthma
Source: Front Allergy. 2021 May 17;2:656886. doi: 10.3389/falgy.2021.656886 (PMC8974720; doi:10.3389/falgy.2021.656886)
Supplement: Supplementary file 1 [file Data_Sheet_1.docx]

**
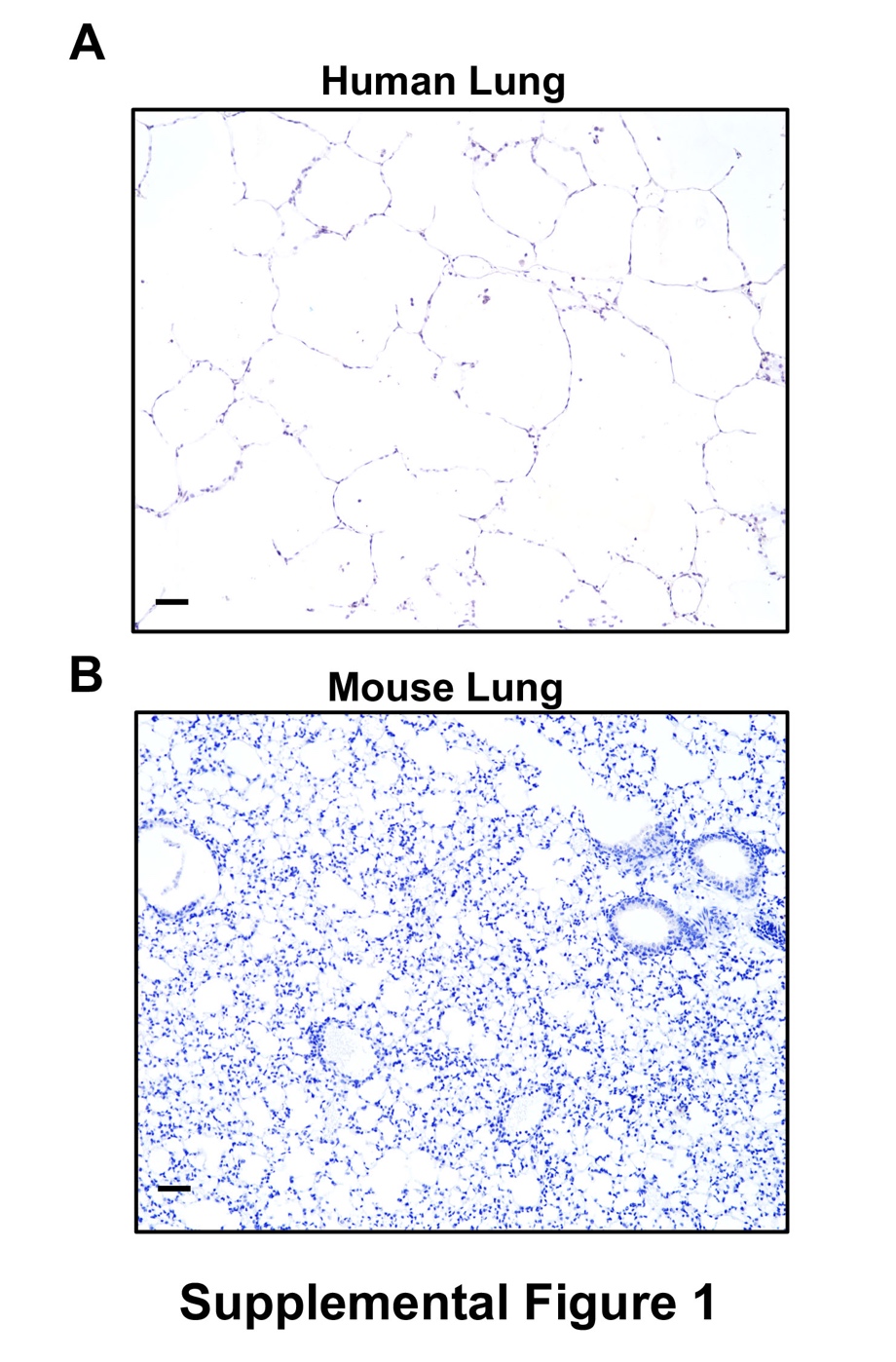
**

**Supplemental Figure 1.** IHC staining of (A) human and (B) mouse lung sections with isotype control antibodies. The sections were stained with isotype control IgG antibodies followed by incubation with the secondary antibody. The slides were washed, treated with the DAB substrate and H&E (counter stain) and visualized using a light microscope. A representative image of a slide is shown. Scale bar=100 μm.
