# Supplementary figures and images for: G Protein-Coupled Receptor Kinase 2 (GRK2) Regulates T Cell Response in a Murine Model of House Dust Mite-Induced Asthma
Source: Front Allergy. 2021 May 17;2:656886. doi: 10.3389/falgy.2021.656886 (PMC8974720; doi:10.3389/falgy.2021.656886)

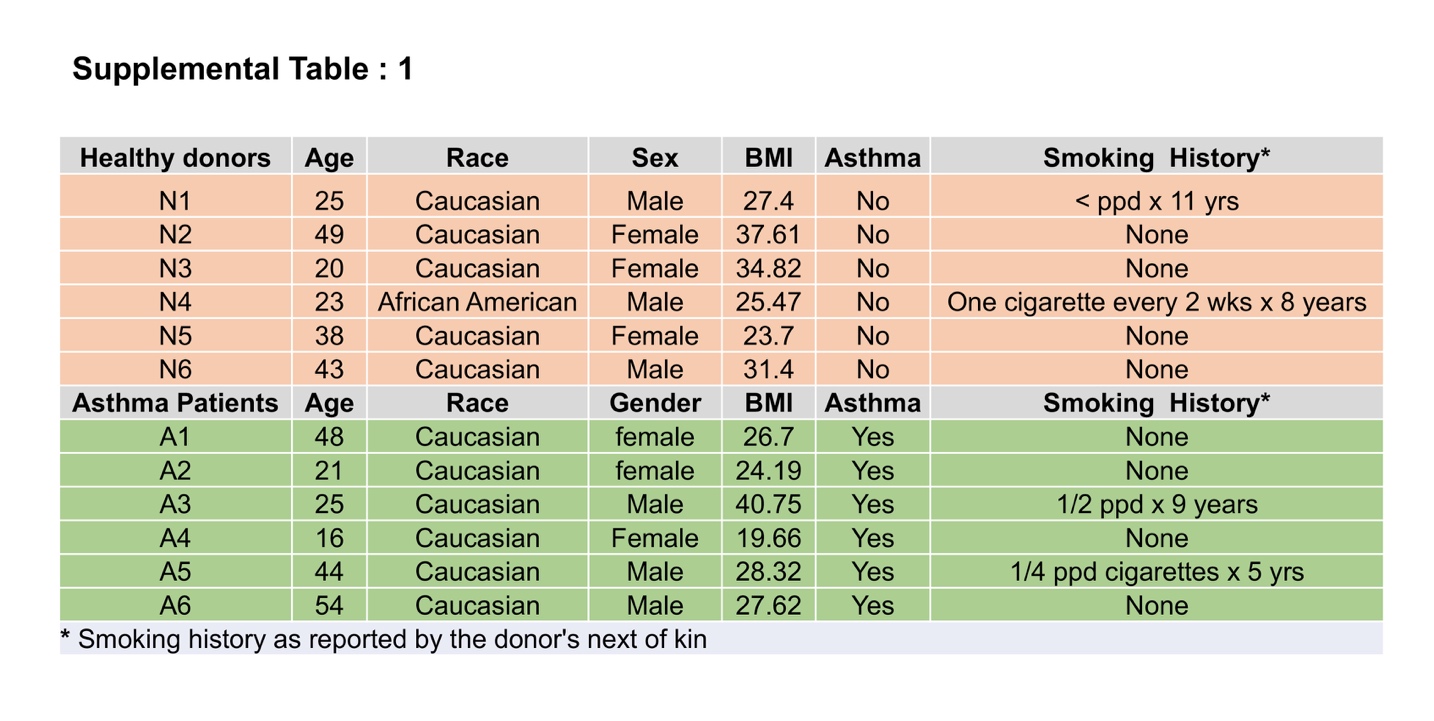

Supplement: Supplementary file 2 [file Table_1.docx]
